# Behaviors Characteristic of Autism Spectrum Disorder in Older Adults With Cognitive Impairment

**DOI:** 10.1093/geroni/igaa057.506

**Published:** 2020-12-16

**Authors:** Elizabeth Rhodus, Justin Barber, Erin Abner, Shani Bardach, Graham Rowles, Gregory Jicha

**Affiliations:** 1 University of Kentucky, Richmond, Kentucky, United States; 2 University of Kentucky, Lexington, Kentucky, United States; 3 University of Kentucky, Lexington, United States

## Abstract

Autism spectrum disorder (ASD) is commonly recognized by the time of adolescence, but is poorly understood in older adults. The possibility of late-life emergence of ASD has been poorly explored. In order to investigate late-life emergence of behaviors characteristic of ASD in MCI and AD, we surveyed caregivers of 142 older adults with neurodegenerative cognitive impairment using the Gilliam Autism Rating Scale-2. Participants with high autism index ratings (Autism ‘Possible/Very Likely’, n=23) reported significantly (statistically and clinically) younger age at onset of cognitive impairment than those who scored in the Autism ‘Unlikely’ range (n=119): 71.14±10.9 vs. 76.65±8.25 (p = 0.034). Additionally, those in Autism ‘Possible/Very Likely’ group demonstrated advanced severity of cognitive impairment, indicated by Clinical Dementia Rating Scale Sum of Boxes scores. Data demonstrate that ASD behaviors may appear de novo of degenerative dementia and such behaviors are more prevalent in those with early onset dementia. Further work elucidating a connection between ASD and dementia could shed light on subclinical forms of ASD, identify areas of shared neuroanatomic involvement between ASD and dementias, and provide valuable insights that might hasten the development of therapeutic strategies.

